# A Comparison of Two Methods for Quantifying Soil Organic Carbon of Alpine Grasslands on the Tibetan Plateau

**DOI:** 10.1371/journal.pone.0126372

**Published:** 2015-05-06

**Authors:** Litong Chen, Dan F. B. Flynn, Xin Jing, Peter Kühn, Thomas Scholten, Jin-Sheng He

**Affiliations:** 1 Key Laboratory of Adaptation and Evolution of Plateau Biota, Northwest Institute of Plateau Biology, Chinese Academy of Sciences, Xining, Qinghai, People’s Republic of China; 2 Department of Ecology, College of Urban and Environmental Sciences, and Key Laboratory for Earth Surface Processes of the Ministry of Education, Peking University, Beijing, People’s Republic of China; 3 Department of Geosciences, Physical Geography and Soil Science, University of Tuebingen, Tuebingen, Germany; Institute of Botany, CHINA

## Abstract

As CO_2_ concentrations continue to rise and drive global climate change, much effort has been put into estimating soil carbon (C) stocks and dynamics over time. However, the inconsistent methods employed by researchers hamper the comparability of such works, creating a pressing need to standardize the methods for soil organic C (SOC) quantification by the various methods. Here, we collected 712 soil samples from 36 sites of alpine grasslands on the Tibetan Plateau covering different soil depths and vegetation and soil types. We used an elemental analyzer for soil total C (STC) and an inorganic carbon analyzer for soil inorganic C (SIC), and then defined the difference between STC and SIC as SOC_CNS_. In addition, we employed the modified Walkley-Black (MWB) method, hereafter SOC_MWB_. Our results showed that there was a strong correlation between SOC_CNS_ and SOC_MWB_ across the data set, given the application of a correction factor of 1.103. Soil depth and soil type significantly influenced on the recovery, defined as the ratio of SOC_MWB_ to SOC_CNS_, and the recovery was closely associated with soil carbonate content and pH value as well. The differences of recovery between alpine meadow and steppe were largely driven by soil pH. In addition, statistically, a relatively strong correlation between SOC_CNS_ and STC was also found, suggesting that it is feasible to estimate SOC_CNS_ stocks through the STC data across the Tibetan grasslands. Therefore, our results suggest that in order to accurately estimate the absolute SOC stocks and its change in the Tibetan alpine grasslands, adequate correction of the modified WB measurements is essential with correct consideration of the effects of soil types, vegetation, soil pH and soil depth.

## Introduction

In recent years, as atmospheric CO_2_ concentrations continue to rise and associated global climate change increasingly becomes a concern, considerable research effort has been put into the feedbacks between the carbon (C) cycle and climate at regional to global scales [1–4]. Soils represent the largest stock of organic C, holding approximately 1,500 Pg (10^15^ g) C in 0–100 cm [5], and even minor changes in soil organic C (SOC) pools can impact on the global C cycle [6]. Due to its importance in the feedbacks between the C cycle and climate [7], considerable effort has been put into accurately quantifying SOC pools and their dynamics over time [8–13]. Unfortunately, a range of different methods have been used for determining SOC, and inconsistencies between many past and present studies have limited the comparability of measurements [14]. Thus, to overcome these obstacles, it is essential to compare directly the methods for SOC determination.

Researchers choose their technique for SOC determination by considering the reliability, reproducibility, time-efficiency, cost of equipment or chemicals and the possible environmental risk [15]. Two major analytical techniques, the wet chemical oxidation method [16, 17] and the dry combustion with automated elemental analyzers, have been widely used to measure SOC content over the past 60 years [14, 18]. Because the Walkley-Black (WB) method is rapid and requires minimum equipment compared to other wet or dry combustion methods [19], it has been the most widely reported procedure for the past several decades. However, this approach may lead to widely variable recovery of SOC [15, 20, 21] and brings the risk of using the hazardous chromium-containing dichromate. In contrast, due to its simplicity and accuracy, the dry combustion method has been increasingly used in many parts of the world, such as the Tibetan Plateau [22–24], despite the higher expense of the analyzer and consumables. Indeed, it has been proposed that automated dry combustion is the only reliable, comprehensive method to determine soil C concentration [14], with the added benefit of simultaneous measurement of N and S. The WB method is therefore being progressively replaced by more accurate dry combustion analyses in many countries [14]. However, when evaluating the change of SOC stocks over time where absolute SOC assessments are required, an important issue arises as to how to correctly interpret historic soil analytical results, which most often were obtained by methods based on WB method measurements. Thus, it is necessary to make direct comparisons between SOC content measurements made by these different approaches.

Owing to its cold and relatively humid environment, the Tibetan Plateau accumulates a large amount of SOC, as estimated at 7.4 Pg in the top 100 cm in alpine grasslands [12] using the modified WB method [19]. With recent interests in examining the soil C stocks and its dynamics [12, 25], and the variation in methods for determining to SOC of alpine grasslands on the Tibetan Plateau in some recent studies [12, 22–24], there is a pressing need to quantify the comparability of SOC measures by different methods.

For this study, we collected 712 soil samples from 36 sites of alpine grasslands across the Tibetan Plateau. We used an elemental analyzer for soil total C (STC) and an inorganic analyzer for soil inorganic C (SIC), and then determined the difference between STC and SIC as SOC_CNS_. We then employed a modified wet oxidation (MWB) based on the original method (WB) as described in Walkley and Black [16]. We assessed the influences of sampling depth, vegetation and soil type in order to compare the results between SOC_CNS_ determined by the difference between STC and SIC, and SOC_MWB_ measured with the MWB method. In order to conveniently estimate SOC stocks, we also compared the results between SOC_CNS_ and STC measured with an elemental analyzer.

## Materials and Methods

### Ethics Statement

There were no specific permits required for the described field studies in the alpine grasslands on the Tibetan Plateau. And the research sites are not privately-owned or protected in any way and the field studies did not involve endangered or protected species.

### Soil sampling and analyses

**Fig 1:**
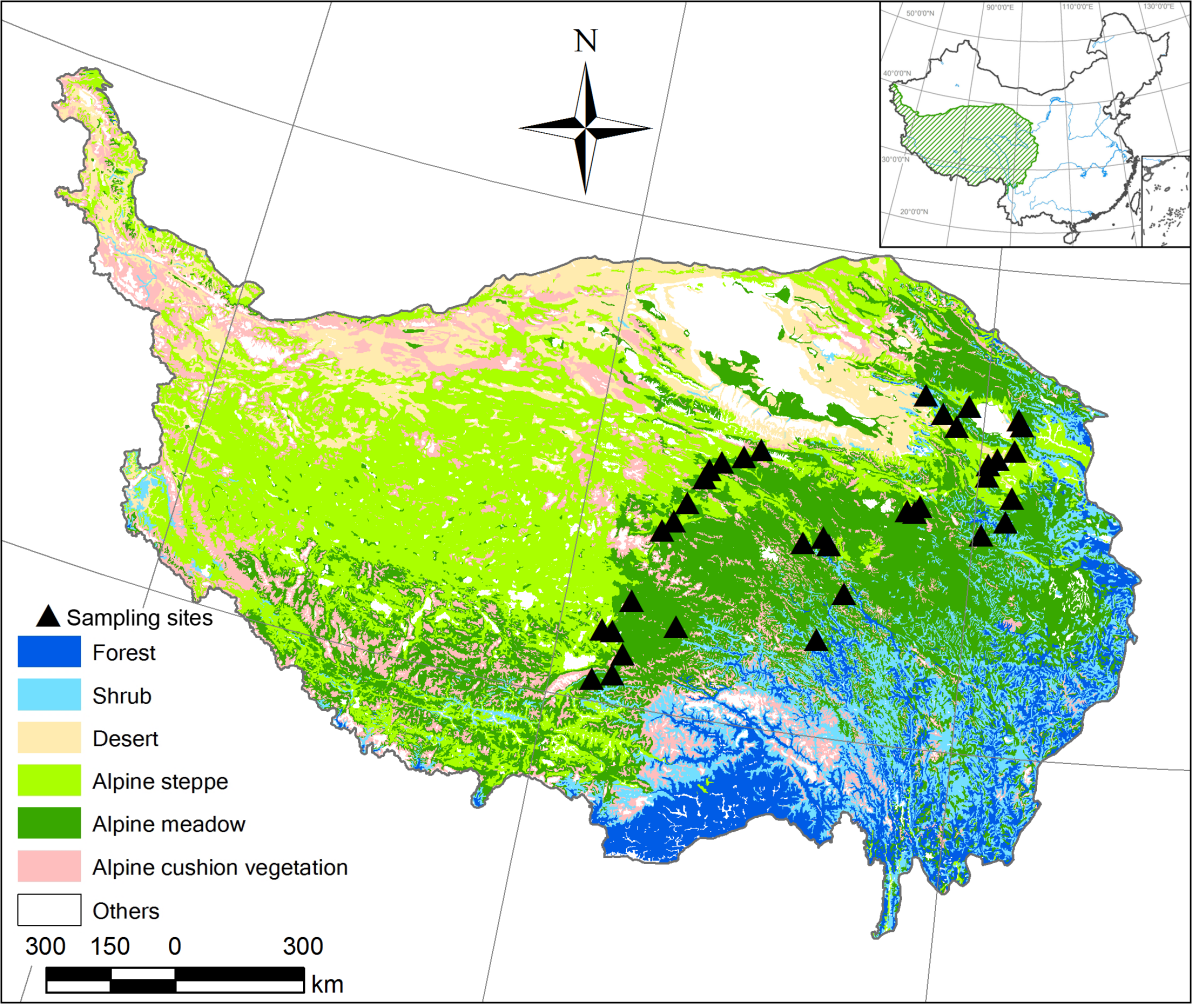
Vegetation map of the 36 sampling sites in the alpine grasslands on the Tibetan Plateau, selected from the Vegetation Map of China [30]. Black triangles represent the sampling sites.

This study was conducted along the transect, ranging from latitudes of 30°27.700′ to 37°16.851′N and longitudes of 91°3.607′ to 101°1.306′E, and elevations from 2938 to 4733 m on the Tibetan Plateau (Fig 1; S1 Table). The plateau has a relatively cold and wet environment with a mean annual temperature range of -9.7 to 6.8°C and mean annual precipitation ranging from 239 to 534 mm [26]. During 2011 (July and August), we collected 712 soil samples from 36 sites (5 profiles per site) in alpine grasslands on the Tibetan Plateau. The sampled sites encompassed two major grassland types of the Tibetan Plateau, alpine meadow and alpine steppe. Alpine meadows are dominated by perennial tussock sedges such as *Kobresia pygmaea*, *Kobresia humilis* and *Kobresia tibetica*, while alpine steppes are dominated by cold-xerophytic graminaceous species such as *Stipa purpurea*, *Stipa subsessiliflora* and *Carex moorcroftiana*; both ecosystem types have extensive distributions [27]. From all profiles at each site, soil samples were collected by hand with a stainless steel core (5 cm in diameter) in four soil increments (0–5, 5–10, 10–20 and 20–30 cm), and 3–8 cores were mixed as a replicate in each soil increments. Sixteen sites were alpine meadow and 20 were alpine steppe (S1 Table). Nine soil types were identified according to the 1:1,000,000 soil map of China [28] and correspondingly 5 soil types based on the World Reference Base for soil resources (WRB) [29] (S1 Table).

Soil samples were taken into the laboratory, air dried, sieved through a 2 mm mesh, handpicked to remove plant detritus, and then ground into a fine powder. The pH of the samples was measured in deionized water at a solution: soil ratio of 2.5:1 with a glass electrode (SevenCompact S220, Mettler-Toledo AG, Switzerland). Carbonate content (calculated as CaCO_3_ content) was determined by a CO_2_ volumetric method by titrating the soil samples with HCl and recording the released CO_2_ (Calcimeter 08.53, Eijkelkamp, Giesbeek, Netherlands).

### SOC analyses after dry heat combustion

SOC content can be determined indirectly from the difference between soil total C (STC) and soil inorganic C (SIC) concentration, measured separately [5]. We measured SIC volumetrically using an inorganic carbon analyzer (Calcimeter 08.53, Eijkelkamp, Giesbeek, Netherlands). STC was by dry combustion using a CNS analyzer (PE 2400 II CHN elemental analyzer, Perkin-Elmer, Boston, Massachusetts, USA) with a combustion temperature of 1150°C and a reduction temperature of 850°C. Soil organic C (SOC_CNS_) was calculated from the difference between STC and SIC:

$${\text{SOC}}_{\text{CNS}}=\text{STC}-\text{SIC}$$

Modified Walkley-Black (MWB) method for SOC

We applied a modified WB method for SOC measurement [16]. The mechanism of this method is to oxidize the organic carbon in the samples to CO_2_ by excessive strong oxidant K_2_Cr_2_O_7_ (using Ag_2_SO_4_ as catalyst), FeSO_4_ is then used to titrated the remnant Cr_2_O_7_^2-^, and the organic carbon content is estimated by the Cr_2_O_7_^2-^ volume consumed during the reaction. A calibration coefficient of 1.10 was used for oxidation efficiency. 0.1–0.5 g soil sample is treated with 5 mL 0.8 M 1/6 K_2_Cr_2_O_7_ standard solution, and then mixed with 5 ml concentrated H_2_SO_4_. The mixture is heated at 170–180°C for 5 minutes with an oil bath furnace, and cooled at room temperature. The solution is transferred into a 250 ml Erlenmeyer flask to keep at 60–80 ml, and unreacted K_2_Cr_2_O_7_ is determined by titrating with 0.2 M FeSO_4_. Soil organic C (SOC_MWB_) content is calculated from the difference in FeSO_4_ used between a blank and a soil solution.

### Calculation of recovery and statistical analyses

The percentage recovery obtained by the MWB method compared to the elemental analyzer method is calculated as:

$$\text{Recovery}=100\times {\text{SOC}}_{\text{MWB}}\;/\;{\text{SOC}}_{\text{CNS}}$$

where SOC_MWB_ = SOC% by the MWB method, and SOC_CNS_ = total organic carbon or SOC% by the elemental analyzer.

In order to consider the effects of vegetation type, soil depth and soil type on the recovery, we performed an analysis of variance (ANOVA) with a Duncan post hoc test. The correction factor was derived for the complete data set, vegetation type, soil depth and soil type using the regression equation passing through the origin for the comparisons of SOC_CNS_, STC and SOC_MWB_.

## Results

**Fig 2:**
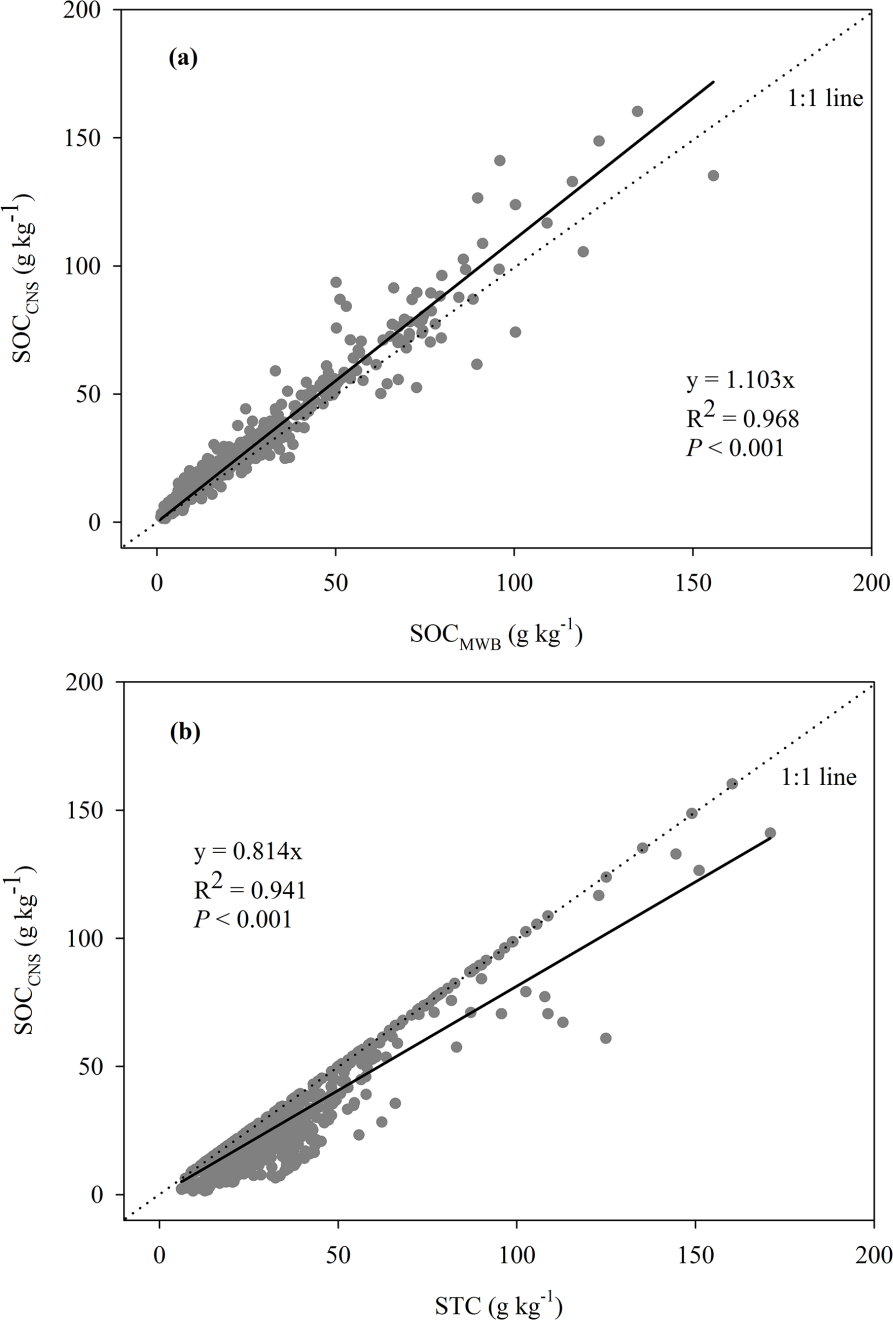
Comparisons of SOC_CNS_ and SOC_MWB_ (a), SOC_CNS_ and STC (b). Solid line is a linear regression passing through the origin. SOC_**CNS**_, the difference between STC and SIC; SOC_**MWB**_, SOC determined by the MWB method.

**Fig 3:**
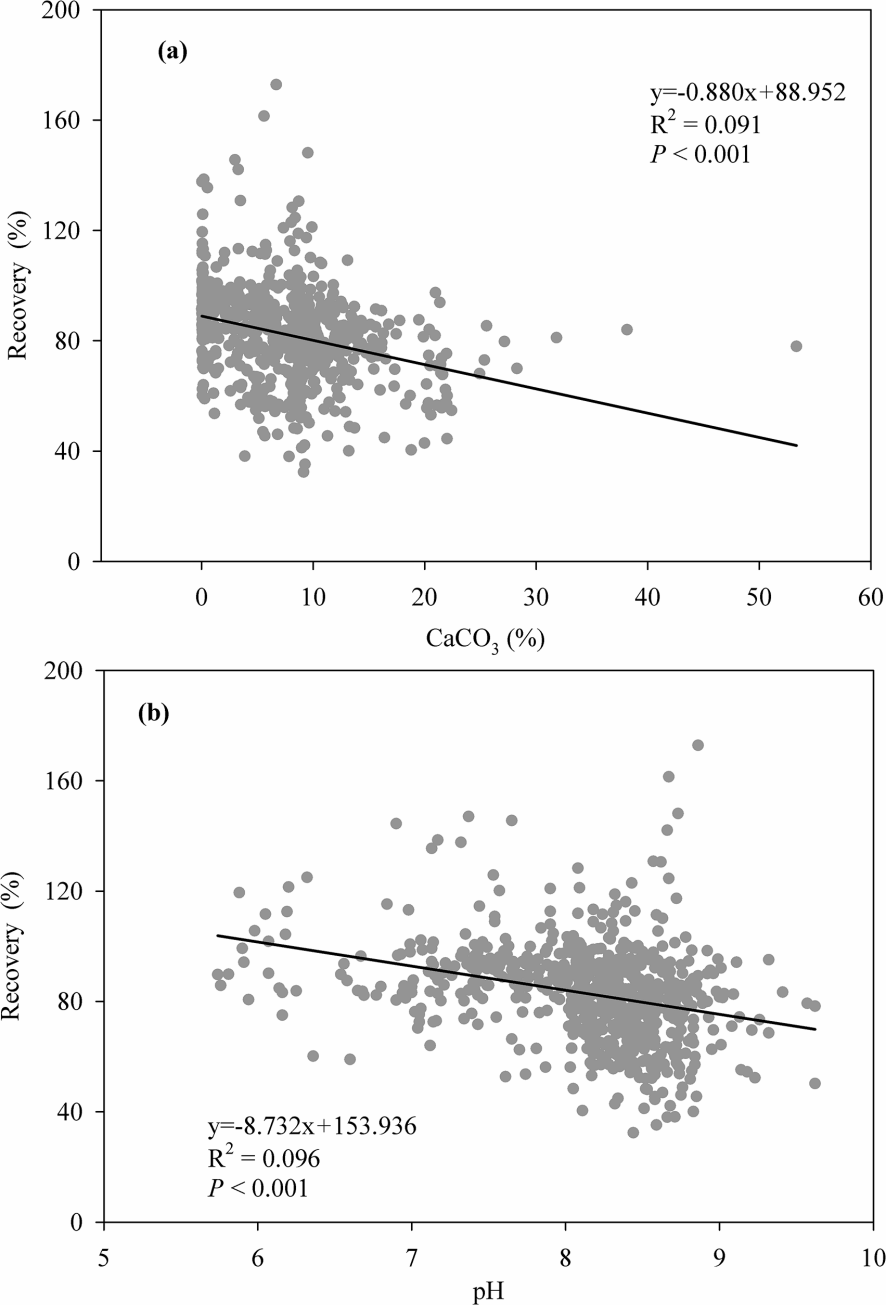
Relationships between the modified Walkey-Black recovery and soil CaCO_3_ content (a) and pH (b) in all 712 soil samples.

In 712 soil samples form the Tibetan Plateau, soil total C determined by the elemental analyzer (STC) varied between 6.10 and 170.90 g kg^-1^, soil inorganic C measured using an inorganic carbon analyzer (SIC) ranged between 0 and 64.00 g kg^-1^, and thus soil organic C (SOC_CNS_) varied between 1.37 and 160.23 g kg^-1^. By contrast, soil organic C determined by the modified Walkley-Black (MWB) method (SOC_MWB_) ranged between 1.12 and 155.71 g kg^-1^. A strong linear relationship between SOC_CNS_ and SOC_MWB_ was found for the complete data set, and greater values of SOC_CNS_ than SOC_MWB_ may be due to more completion oxidation by heat combustion method than the MWB method, which was partly affected by soil CaCO_3_ content and pH (Fig 2A and Fig 3). Therefore, a correction factor of 1.103 is necessary when recalculating SOC stocks determined by the MWB method on this alpine region. Similarly, a relatively strong correlation between SOC_CNS_ and STC was also found (Fig 2B).

**Fig 4:**
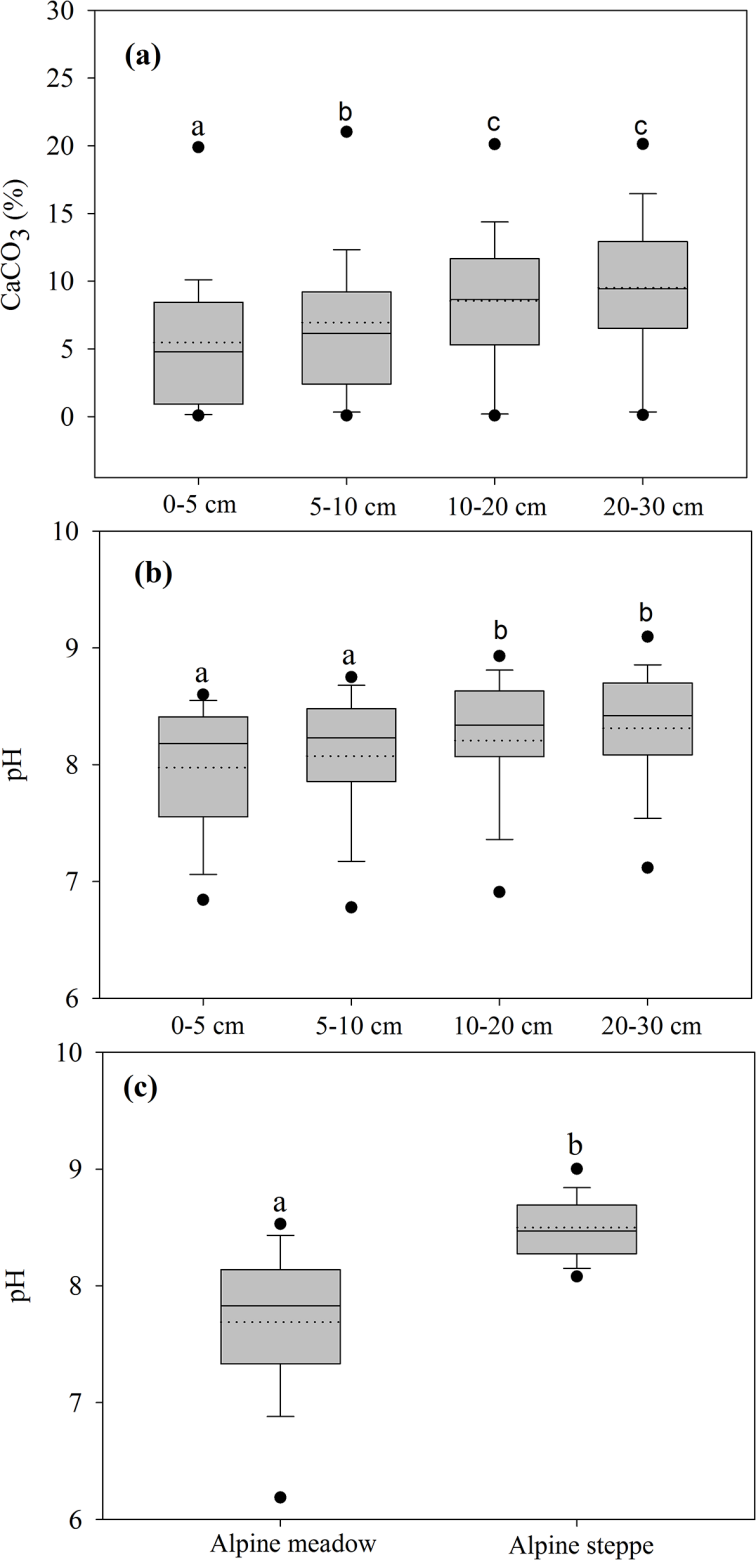
Boxplots of CaCO_3_ content (a) and pH (b) in 0–5 cm, 5–10 cm, 10–20 cm, 20–30 cm Soils, and pH (c) in soils of alpine meadow and steppe. Solied lines within the boxes give the median, dotted lines give the mean, boxes the 25th and 75th percentile. The whiskers are the 5th and 95th percentile and the outliers are closed circles.

The ANOVA showed that soil depth and soil type had significant influences the recovery of SOC measured by the MWB method, and the interaction between vegetation type and soil type also significantly affected this recovery (Table 1). For soil depth, the average recovery was best for 0–5 cm soil (86.3%), then for 5–10 and 10–20 cm soil (82.1% and 83.1%); the least recovery was found for the depth increment 20–30 cm soil (79.8%). The different recoveries among depth increments could be related to soil CaCO_3_ content and pH value because they were significantly lower in the surface soil (e.g. 0–5 cm soil) than other depth increments (Fig 4A and 4B), and both revealed apparent and negative correlations with the recovery (Fig 3). Thus, despite the strong relationship between SOC_CNS_ and SOC_MWB_, the corresponding correction factors of 0–5 cm depth increment were lower than those in other depth increments (Table 2). Similarly, there were also strong correlations between SOC_CNS_ and STC in all depth increments but the strength of correlations became weaker with depth increments (Table 2).

**Table 1:** Results of three-ways ANOVAs on the effects of soil depth, vegetation type, soil type and their interactions on the modified Walkley-Black recovery.

	df	*F*	*P*
**Soil depth**	3	4.345	0.005
**Vegation type**	1	0.291	0.590
**Soil type**	8	11.897	< 0.001
**Soil depth × Vegetation type**	3	0.362	0.780
**Soil depth × Soil type**	24	1.300	0.154
**Vegetation type × Soil type**	1	18.059	< 0.001
**Soil depth × Vegetation type × Soil type**	3	0.721	0.540

**Table 2:** Regressional relationships of SOC and STC determined between by the elemental analyzer and by the modified Walkley-Black (MWB) method in four soil depths on the Tibetan Plateau using a linear regression passing through the origin.

Soil depth (cm)		SOC_CNS_ vs. SOC_MWB_			SOC_CNS_ vs. STC		
	n	Slope	R^2^	*P*	Slope	R^2^	*P*
**0–5**	179	1.054	0.967	<0.001	0.907	0.972	<0.001
**5–10**	180	1.158	0.976	<0.001	0.830	0.954	<0.001
**10–20**	179	1.141	0.970	<0.001	0.728	0.932	<0.001
**20–30**	174	1.161	0.965	<0.001	0.606	0.898	<0.001

SOC_CNS_, the difference between STC and SIC; SOC_MWB_, SOC determined by the MWB method.

**Fig 5:**
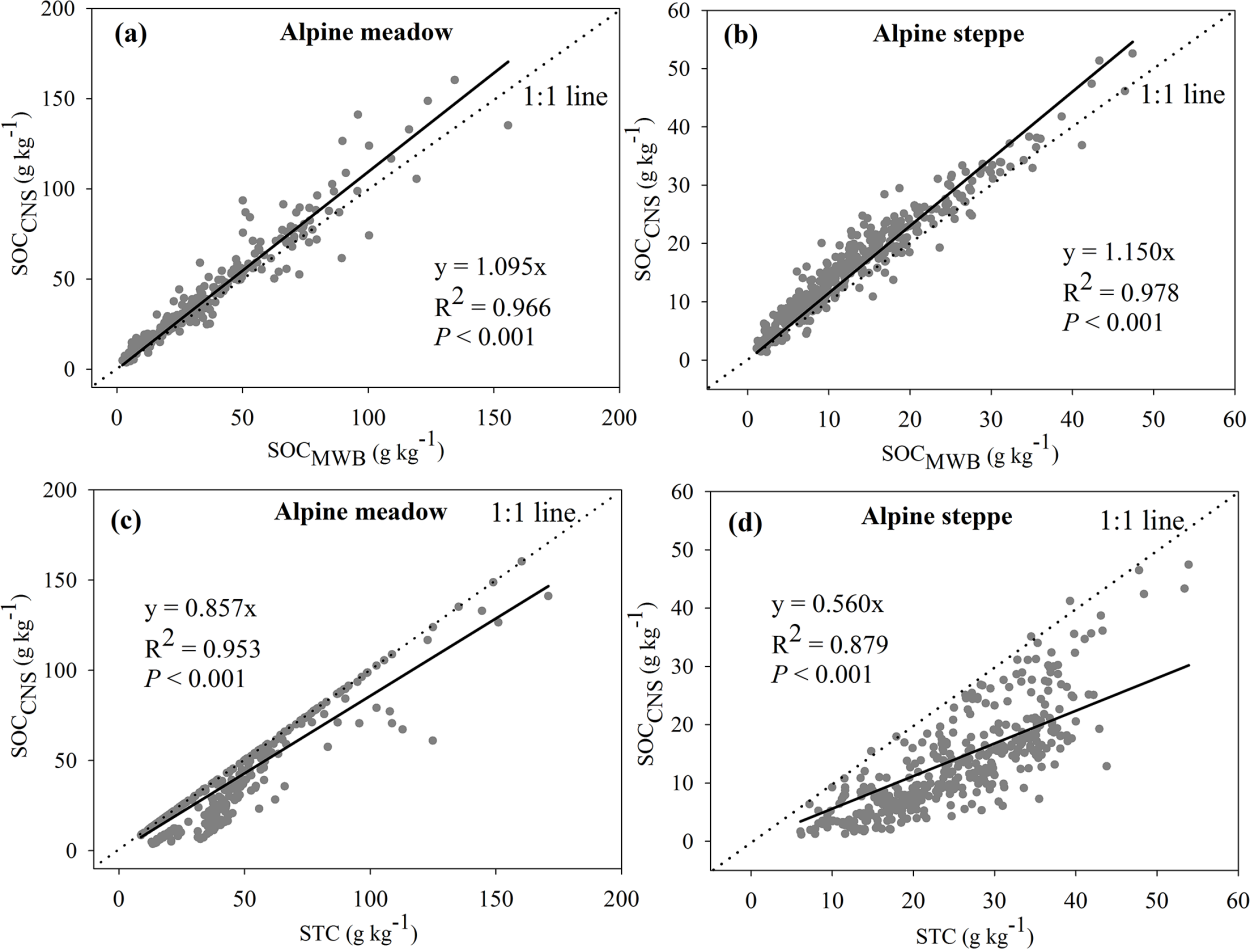
Comparisons of SOC_CNS_ and SOC_MWB_, SOC_CNS_ and STC for alpine medow (a, c) and steppe (b, d). Solid line is a linear regression passing through the origin. SOC_**CNS**_, the difference between STC and SIC; SOC_**MWB**_, SOC determined by the MWB method.

Although vegetation type did not significantly influence recovery, the recovery in alpine meadow soils (85.2%) tended to be higher by about 5% than that of alpine steppe soils (80.8%). The different recovery among the two grassland types could be related to soil pH value, as alpine meadow soils are significantly more acidic than those in alpine steppes (Fig 4C). Despite the strong relationships between SOC_CNS_ and SOC_MWB_, the corresponding correction factor in alpine steppe soil was higher than that in alpine meadow soils (Fig 5A and 5B). Strong correlations between SOC_CNS_ and STC were detectable between alpine meadow and alpine steppe, and the correlations between them in alpine steppe was much weaker than that in alpine meadow (Fig 5C and 5D).

**Fig 6:**
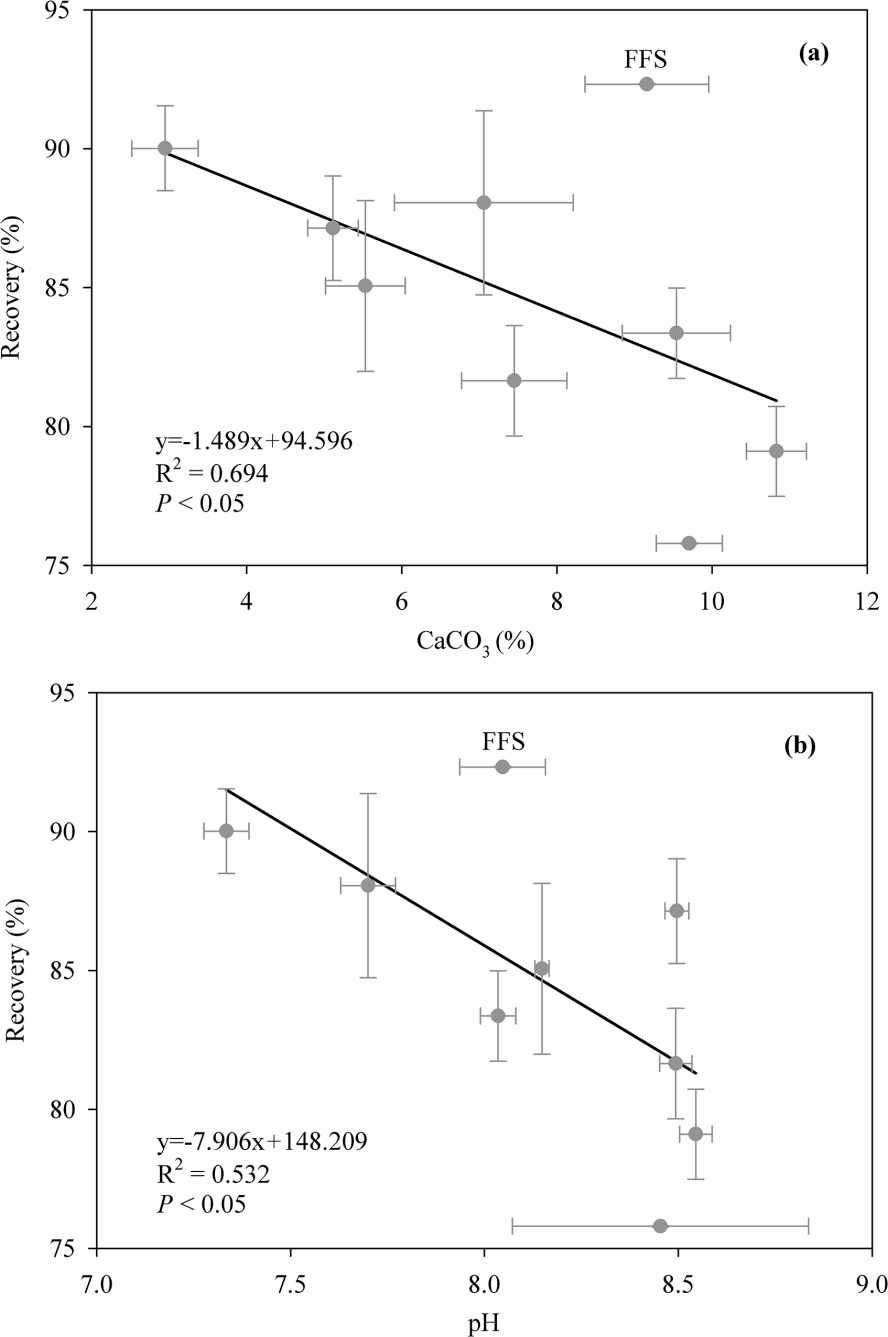
Relationships between CaCO_3_ content (±SE, a) and pH (±SE, b) and the modified Walkey-Black average recovery (±SE) in 9 soil types (accroding to the GSCC, Genetic Soil Classification of China) on the Tibetan Plateau. Equations, linear fits (R^2^) and significant levels (*P*) were obtained when ‘frigid frozen soils (FFS)’ was excluded from the analyses.

In nine soil types on the Tibetan Plateau, frigid calcic soils, one of main soil types, had the lowest average recovery (75.8%), while the frigid frozen soil and felty soil had the relatively higher average recovery (92.3% and 90.1%, respectively). Except frigid frozen soils, the average recovery in other 8 soil types was significantly affected by CaCO_3_ content and pH value (Fig 6). Therefore, the corresponding correction factor differed among the nine soil types despite the significant relationships between SOC_CNS_ and SOC_MWB_ in these soil types (Table 3). The correction factor varied from 1.047 in felty soils to 1.215 in frigid calcic soils. Similarly, the correction factor also differed among soil types based the WRB, ranging from 1.079 in Cambisols to 1.215 in frigid calcic soils (S2 Table). Addtionally, there were strong correlations between SOC_CNS_ and STC in these soil types, but the strength of correlations differed among these soil types (Table 3; S2 Table).

**Table 3:** Regressional relationships of SOC and STC determined between by the elemental analyzer and by the modified Walkley-Black (MWB) method in 9 soil types (accroding to the GSCC, Genetic Soil Classification of China) on the Tibetan Plateau using a linear regression passing through the origin.

Soil type		SOC_CNS_ vs. SOC_MWB_			SOC_CNS_ vs. STC		
	n	Slope	R^2^	*P*	Slope	R^2^	*P*
**Felty soils**	139	1.047	0.975	<0.001	0.963	0.987	<0.001
**Meadow soils**	80	1.089	0.988	<0.001	0.823	0.944	<0.001
**Frigid frozen soils**	20	1.091	0.986	<0.001	0.769	0.973	<0.001
**Castanozems**	100	1.106	0.980	<0.001	0.802	0.970	<0.001
**Dark felty soils**	36	1.131	0.952	<0.001	0.968	0.917	<0.001
**Cold calcic soils**	39	1.135	0.984	<0.001	0.745	0.952	<0.001
**Chernozems**	20	1.165	0.975	<0.001	0.715	0.982	<0.001
**Brown pedocals**	38	1.213	0.986	<0.001	0.596	0.986	<0.001
**Frigid calcic soils**	240	1.215	0.983	<0.001	0.672	0.913	<0.001

SOC_CNS_, the difference between STC and SIC; SOC_MWB_, SOC determined by the MWB method.

## Discussion

Precise determination of soil organic C (SOC) is of critical importance for detection of C sequestration or emission potential induced by global environmental changes, such as warming, nitrogen deposition, elevated CO_2_ concentrations and land use change. Many methods are currently available, each with advantages and disadvantages with regards to accuracy, expense and convenience [14, 18]. Owing to its low cost and minimal requirements in laboratory equipment, the Walkley-Black (WB) procedure is still used widely throughout the world to measure SOC content [14]. However, the largest limitation in the WB method is highly variable recovery percentage, and thus a correction factor is often applied to determine the total SOC content for a soil sample. Originally, Walkley and Black [16] decided that the recovery was on average 76% and thus a correction factor of 1.32 was introduced for quantifying the total SOC content of a soil sample. Using the original WB method, recent studies quantified the correction factor as 1.51–1.77 in 475 samples of silt loam and sandy soil in northern Belgium [15], and 1.58 in 542 soil samples from temperate lowland forests [21]. However, another showed that the original WB method produced approximately 100% recovery for SOC in most Tasmanian soils [31]. In addition, several studies suggested that the modified WB techniques which involved extensive heating did not require a correction factor [32–35], whereas one study indicated that other methods which involved minimal heating required a small correction factor of 1.15 [34]. Based on 712 soil samples in alpine grasslands on the Tibetan Plateau, we quantified a correction factor of 1.103, which is comparable to these above-mentioned studies for the modified technique. The correction factor of 1.103 obtained from soils of alpine grasslands in the Tibetan Plateau may be applied to other high-latitude tundra ecosystem (e. g. Arctic tundra) because they share many features, such as permafrost-influenced soil, high soil carbon storage, vegetation community (e. g. tussock sedges and cold-xerophytic graminoids) and cold climatic condition with a long non-growing season [12, 27, 36–38].

Although no correlation of the recovery rate was found with soil pH and carbonate content [18], we found that soil pH and carbonate content had significantly impacts on the recovery despite their influences were relatively weak (only 9.6% and 9.1%). Additionally, we also found that the recovery and thus the correction factor were affected significantly by soil depth, vegetation and soil type.

SOC from the deeper horizons is widely considered to be the most easily oxidized, probably because of accumulation of fulvic acids and because there are fewer plant residues [18]. Therefore, the recovery generally increases with increasing sampling depth [39]. However, there was not a clear trend between recovery and soil sampling depth [15]. And even no correlation was also observed between the recovery and soil sampling depth in other studies [40–42]. In contrast to these studies, we found that the upper depth increment had a better recovery than the deeper depth increments of alpine grasslands on the Tibetan Plateau. Soil pH and carbonate content may be partly responsible for the trend between the recovery and depth because there were higher pH values and carbonate content in the deeper depth increments (Fig 4A and 4B). Another reason for a decreasing recovery rate with increasing soil depth could be higher amount of occluded particulate and mineral associated organic matter in subsoils of the Tibetan Plateau compared to the topsoil [23].

Related to effects of vegetation type on SOC, Díaz-Zorita [40] reported that the recovery was less under pasture than under other cultivation systems on a loam soil in the subhumid Argentinean Pampa (0–15 cm). This could be explained by the presence of a high percentage of recalcitrant soil organic matters (SOM, e.g., phenolics and lignin compounds). These recalcitrant SOM are resistant to oxidation at temperatures obtained with H_2_SO_4_ in the WB analytical procedure. A few studies also demonstrated a poorer recovery in forests compared to grasslands, because tree species have relatively higher leaf lignin than grass species [15, 43]. In the present study, we found that alpine meadow had a greater recovery than alpine steppe on the Tibetan Plateau. The difference could be attributed to the fact that compared to alpine meadow, alpine steppe vegetation consists mainly of the lignin-rich graminaceous species, such as *Stipa purpurea*, *Stipa subsessiliflora* and *Carex moorcroftiana* [27], and consequently soils in alpine steppe contain a high percentage of recalcitrant lignin compounds. Since leaf phenolics in alpine meadow were higher than that in alpine steppe [44], soil phenolics may be not responsible for the different recovery between alpine meadow and alpine steppe in our study. In addition, our results demonstrated that soil pH in alpine meadow soils was significantly lower than that in alpine steppe soils (Fig 4C), soil pH therefore was another probable factor resulted in such a recovery difference between them.

Regarding the effect of soil type on the recovery, Allison [34] proposed that the large heterogeneity in the recovery of organic C was due to differences in soil formation and subsequently soil types. Accordingly, our results indicated that the recovery differed greatly over the nine soil types tested (75.8–92.3%), and the correction factor varied from 1.047 in felty soils to 1.215 in frigid calcic soils. Without considering frigid frozen soils, the greater difference in the recovery among other 8 soil types could be associated closely with soil pH and carbonate content because the recovery of organic C in these soil types were significantly and negatively correlated to soil pH and carbonate content (Fig 6).

SOC was commonly measured by the elemental analyzer after removing the carbonate with adding acid. Currently, however, more and more researchers indirectly determined the SOC content as we did in this study, that is, the difference between STC and SIC measured separately [22–24, 45]. In this study, we also explored the relationships between SOC_CNS_ and STC, and found a strong correlation between them (Fig 2B), whereas the strong correlation was affected by sampling depth, vegetation and soil types (Tables 2 and 3; Fig 5). Therefore, although it is feasible to estimate SOC stocks through the STC, we must caution the effect of soil depth, vegetation and soil types.

## Conclusions

Based on 712 soil samples from 36 sites of alpine grasslands on the Tibetan Plateau, we made direct comparison of SOC measurements from two common techniques, directly from the modified Walkley-Black (MWB) method and indirectly by an elemental analyzer. The comparison indicate that in order to accurately estimate the absolute soil stock and its change over time, adequate correction of the soil SOC data obtained from the MWB method is crucial, however, researchers must consider the influences of soil depth, vegetation and soil types. Additionally, our comparison also suggest that it is feasible to estimate SOC stocks through the STC data across the Tibetan grasslands.

## Supporting Information

S1 Table Description of 36 sites across the Tibetan Plateau grasslands where 712 soil samples were collected. Data for latitude, longitude and altitude were obtained with Magellan GPS Field PROV (Magellan System Corporation, San Dimas, CA, USA). GSCC, Genetic Soil Classification of China; WRB, World Reference Base for soil resources.
(PDF)

S2 Table Regressional relationships of SOC and STC determined between by the elemental analyzer and by the modified Walkley-Black (MWB) method in 5 soil types (accroding to the WRB, World Reference Base for soil resources) on the Tibetan Plateau using a linear regression passing through the origin. SOC_CNS_, the difference between STC and SIC; SOC_MWB_, SOC determined by the MWB method.
(PDF)
