# Supplementary material for: A Comparison of Two Methods for Quantifying Soil Organic Carbon of Alpine Grasslands on the Tibetan Plateau
Source: PLoS One. 2015 May 6;10(5):e0126372. doi: 10.1371/journal.pone.0126372 (PMC4422439; doi:10.1371/journal.pone.0126372)
Supplement: S1 Table — Data for latitude, longitude and altitude were obtained with Magellan GPS Field PROV (Magellan System Corporation, San Dimas, CA, USA). GSCC, Genetic Soil Classification of China; WRB, World Reference Base for soil resources. (PDF) [file pone.0126372.s001.pdf]

S1 Table. Description of 36 sites across the Tibetan Plateau grasslands where 712 soil samples were collected. Data for latitude, longitude and altitude were obtained with Magellan GPS Field PROV (Magellan System Corporation, San Dimas, CA, USA). GSCC, Genetic Soil Classification of China; WRB, World Reference Base for soil resources.

**Site**	**Longitude (°N)**	**Latitude (°E)**	**Altitude (m)**	**Vegetation type**	**Soil type-GSCC**	**Soil type-WRB**
1	100°53.432'	36°19.221'	3282	alpine steppe	Cold calcic soils	Cambisols
2	100°27.422′	36°07.280′	2938	alpine steppe	Castanozems	Kastanozems
3	100°13.207′	35°59.973′	3080	alpine steppe	Castanozems	Kastanozems
4	100°13.498′	35°45.724′	3193	alpine steppe	Castanozems	Kastanozems
5	100°55.751′	35°21.083′	3366	alpine steppe	Chernozems	Chernozems
6	100°49.393′	34°51.414′	3663	alpine meadow	Felty soils	Cambisols
7	100°13.176′	34°32.108′	3728	alpine meadow	Felty soils	Cambisols
8	98°34.592′	34°59.056′	4305	alpine steppe	Frigid calcic soils	Calcisols
9	98°27.071′	34°51.325′	4230	alpine steppe	Frigid calcic soils	Calcisols
10	96°55.205′	33°01.548′	3825	alpine meadow	Dark felty soils	Cambisols
11	96°23.657′	32°00.170′	4177	alpine meadow	Dark felty soils	Cambisols
12	95°42.232′	33°56.836′	4152	alpine meadow	Meadow soils	Cambisols
13	96°22.252′	33°58.190′	4224	alpine meadow	Meadow soils	Cambisols
14	96°12.235′	34°06.083′	4366	alpine meadow	Meadow soils	Cambisols
15	98°15.121′	34°52.502′	4243	alpine steppe	Frigid calcic soils	Calcisols
16	101°01.306′	36°51.842′	3066	alpine steppe	Castanozems	Kastanozems
17	100°55.340′	36°58.825′	3130	alpine steppe	Castanozems	Kastanozems
18	99°34.223′	37°08.267′	3241	alpine steppe	Meadow soils	Cambisols
19	98°54.732′	36°56.347′	3297	alpine steppe	Cold calcic soils	Cambisols

20	98°23.293′	37°16.851′	3425	alpine steppe	Cold brown calcic soils	Cambisols
21	94°16.972′	35°42.446′	4374	alpine meadow	Frigid frozen soils	Leptosols
22	93°51.934′	35°30.723′	4598	alpine steppe	Frigid calcic soils	Calcisols
23	93°19.314′	35°19.557′	4539	alpine meadow	Frigid calcic soils	Calcisols
24	93°02.413′	35°07.700′	4733	alpine steppe	Frigid calcic soils	Calcisols
25	92°56.901′	34°56.520′	4555	alpine steppe	Frigid calcic soils	Calcisols
26	92°37.389′	34°22.662′	4625	alpine steppe	Frigid calcic soils	Calcisols
27	92°21.163′	33°57.198′	4619	alpine meadow	Frigid calcic soils	Calcisols
28	92°06.048′	33°43.622′	4697	alpine steppe	Frigid calcic soils	Calcisols
29	91°40.592′	32°11.645′	4632	alpine meadow	Felty soils	Cambisols
30	91°20.024′	31°30.287′	4664	alpine meadow	Felty soils	Cambisols
31	91°04.004′	31°29.796′	4608	alpine steppe	Frigid calcic soils	Calcisols
32	92°52.145′	31°49.888′	4284	alpine meadow	Felty soils	Cambisols
33	91°41.109′	31°04.469′	4702	alpine meadow	Felty soils	Cambisols
34	91°32.196′	30°37.291′	4542	alpine meadow	Felty soils	Cambisols
35	91°03.607′	30°27.007′	4256	alpine meadow	Frigid calcic soils	Calcisols
36	99°18.567′	36°42.040′	3137	alpine steppe	Cold brown calcic soils	Cambisols
