# Supplementary material for: A Comparison of Two Methods for Quantifying Soil Organic Carbon of Alpine Grasslands on the Tibetan Plateau
Source: PLoS One. 2015 May 6;10(5):e0126372. doi: 10.1371/journal.pone.0126372 (PMC4422439; doi:10.1371/journal.pone.0126372)
Supplement: S2 Table — SOCCNS, the difference between STC and SIC; SOCMWB, SOC determined by the MWB method. (PDF) [file pone.0126372.s002.pdf]

S2 Table. Regressional relationships of SOC and STC determined between by the elemental analyzer and by the modified Walkley-Black (MWB) method in 5 soil types (accroding to the WRB, World Reference Base for soil resources) on the Tibetan Plateau using a linear regression passing through the origin. SOC_{CNS}, the difference between STC and SIC; SOC_{MWB}, SOC determined by the MWB method.

Soil type		SOC_{CNS} vs. SOC_{MWB}			SOC_{CNS} vs. STC		
	n	Slope	R^2	*P*	Slope	R^2	*P*
Cambisols	332	1.079	0.972	<0.001	0.878	0.959	<0.001
Leptosols	20	1.091	0.986	<0.001	0.769	0.973	<0.001
Kastanozems	100	1.106	0.980	<0.001	0.802	0.970	<0.001
Chernozems	20	1.165	0.975	<0.001	0.715	0.982	<0.001
Calcisols	240	1.215	0.983	<0.001	0.672	0.913	<0.001
